# A New Discovery

**Published:** 1883-02

**Authors:** 


*A New Discovery.*—Dr. G. Bœck says: If potatoes are peeled and treated with eight parts sulphuric acid and one hundred parts of water, and then dried and pressed, a mass is obtained very like celluloid, and which can be used instead of meerchaum or ivory. It is not stated whether the invention is protected by a patent or not.

